# Case report: A challenging case of severe Cushing’s syndrome in the course of metastatic thymic neuroendocrine carcinoma with a synchronous adrenal tumor

**DOI:** 10.3389/fendo.2024.1399930

**Published:** 2024-06-14

**Authors:** Lukasz Dzialach, Agnieszka Wojciechowska-Luzniak, Maria Maksymowicz, Przemysław Witek

**Affiliations:** ^1^ Department of Internal Medicine, Endocrinology and Diabetes, Medical University of Warsaw, Warsaw, Poland; ^2^ Department of Pathology and Laboratory Diagnostics, Maria Sklodowska-Curie National Research Institute of Oncology, Warsaw, Poland

**Keywords:** adrenal tumor, cortisol, ectopic ACTH syndrome, etomidate, thymic neuroendocrine carcinoma

## Abstract

Ectopic ACTH syndrome (EAS) remains one of the most demanding diagnostic and therapeutic challenges for endocrinologists. Thymic neuroendocrine tumors account for 5%–10% of all EAS cases. We report a unique case of a 31-year-old woman with severe EAS caused by primary metastatic combined large-cell neuroendocrine carcinoma and atypical carcinoid of the thymus. The patient presented with severe hypercortisolemia, which was successfully controlled with continuous etomidate infusion. Complex imaging initially failed to detect thymic lesion; however, it revealed a large, inhomogeneous, metabolically active left adrenal mass infiltrating the diaphragm, suspected of primary disease origin. The patient underwent unilateral adrenalectomy, which resulted in hypercortisolemia resolve. The pathology report showed an adenoma with adrenal infarction and necrosis. The thymic tumor was eventually revealed a few weeks later on follow-up imaging studies. Due to local invasion and rapid progression, only partial resection of the thymic tumor was possible, and the patient was started on radio- and chemotherapy.

## 1 Introduction

Endogenous Cushing’s syndrome (CS) is a rare endocrine condition caused by excess cortisol production with an annual incidence of 0.2–5 cases per million people (1). Adrenocorticotropin (ACTH) hypersecretion of nonpituitary tumors leading to ectopic ACTH syndrome (EAS) accounts for 9%–18% of ACTH-dependent CS cases (1, 2) and represents one of the most common paraneoplastic syndromes (3, 4). Neuroendocrine tumors (NETs) of various locations, degrees of histological differentiation, and aggressiveness potential can lead to EAS; however, most frequently, they derive from the foregut, with the well-differentiated bronchial NET being the most common one in recent series (5, 6). NETs of the thymus (NETTs) represent up to 5% of all thymic tumors, with an incidence of 0.02 per 100,000 people per year in the Caucasian population (7, 8). Up to 50% of the hormonally active NETTs present with ACTH hypersecretion (8) that account for 5%–10% of EAS cases (5, 9). They usually behave aggressively with regional invasion and early distant metastases and lead to the rapid development of severe hypercortisolism (SH), which worsens the initial poor prognosis (8–11).

Herein, we present a unique case of a patient with EAS caused by a primary metastatic, ACTH-secreting thymic large-cell neuroendocrine carcinoma (LCNEC) with an atypical carcinoid (AC) component with rapid progression, which initially failed to be visualized in imaging studies. Moreover, the diagnostic process was even more difficult because of the co-presence of an adrenal lesion suspected of malignancy on imaging studies and to be the primary origin of the disease.

## 2 Case report

In April 2019, a 31-year-old previously healthy woman presented to the Emergency Department with a 3-week history of progressing fatigue, muscle weakness, exercise intolerance, headaches, progressive hypertension, generalized swelling, polyuria, polydipsia, and nycturia. Due to the reported symptoms, the patient had previously consulted a family doctor, who initiated oral potassium supplementation because of hypokalemia (2.8 mmol/L) found in basic laboratory tests. On physical examination, the patient presented with significant peripheral pitting edema, high blood pressure (170/100 mmHg), tachycardia (170 beats/minute), and acne lesions on the face, back, and chest. The initial laboratory tests at the Emergency Department showed the following: leukocytosis (13.95 × 10^9^/L) with neutrophilia (12.98 × 10^9^/L) and lymphopenia (0.27 × 10^9^/L), hypochloremic metabolic alkalosis (pH 7.52; HCO_3_^−^, 38.3 mmol/L; and Cl^−^, 91 mmol/L), hyperglycemia (478 mg/dL), and profound hypokalemia (2.2 mmol/L). Initial laboratory findings are summarized in **Table 1A**.

**Table 1A:** Summary of initial laboratory test performed at Emergency Department.

Parameter	Value	Reference range
Blood morphology		
Leukocytes (10^9^/L)	13.95	4.0–10.0
Neutrophils (10^9^/L)	12.98	1.9–8.0
Basophils (10^9^/L)	0.03	0.0–0.2
Eosinophils (10^9^/L)	0.0	0.05–0.5
Lymphocytes (10^9^/L)	0.27	0.9–4.5
Monocytes (10^9^/L)	0.67	0.1–1.0
Erythrocytes 10^6^/L	4.36	3.5–5.5
Platelets (10^9^/L)	172	150–400
Hemoglobin (g/dL)	12.4	12.0–16.0
Hematocrit (%)	37	35–55
Venous blood gas analysis		
pH	7.52	7.35–7.45
pCO_2_	49.4	35.0–48.0
HCO3^-^	38.3	21.0–27.0
Lactate (mmol/L)	4.4	0.5–2.2
Osmolality (mOsm/kg)	309.7	275–295
Biochemistry		
BUN (mg/dL)	42	15–43
Creatinine (mg/dL)	0.9	0.5–1.0
Glucose (mg/dL)	478	Fasting: 70–99
Sodium (mmol/L)	146	135–145
Potassium (mmol/L)	2.2	3.5–5.1
Chloride (mmol/L)	91	98–106
Total bilirubin (mg/dL)	1.4	0.0–1.2
ALT (U/L)	55	0–33
AST (U/L)	27	0–31
Amylase (U/L)	15	28–100
CRP (mg/dL)	7.9	< 0.5
Coagulogram		
INR	1.01	0.8–1.2
PT (sec)	21.4	23.0–35.0
APTT (sec)	11.4	12.0–16.0

ALT, alanine aminotransferase; APTT, partial thromboplastin time; AST, aspartate aminotransferase; BUN, blood urea nitrogen; CRP, C-reactive protein; HCO_3_^-^, bicarbonate; INR, prothrombin time (international normalized ratio); pCO_2_, partial pressure of carbon dioxide; PT, prothrombin time.

Within the Emergency Department, the patient was considered as a patient with newly diagnosed diabetes and was referred to the Endocrinology Department, where she was started on insulin therapy along with aggressive hypokalemia repletion, antihypertensive treatment, and preventive heparin anticoagulation. Given the overall clinical presentation and resistance to initiated treatment, aggressive CS was quickly suspected. During the first days of hospitalization, the patient also developed agitation with paranoid symptoms; thus, the psychiatrist was consulted, and the patient was additionally started on antipsychotic treatment.

The hormonal evaluation revealed SH with high concentrations of morning (78.2 μg/dL; reference range, 3.7–19.4) and midnight (69.1 μg/dL; reference range, < 5.4) serum cortisol, 24 h urinary free cortisol (UFC) excretion exceeding 65 times the upper reference limit (11,587.5 μg/24 h; reference range, 4.6–176.0), and hyperandrogenemia (testosterone, 6.3 ng/mL; reference range, 0.06–0.8; DHEA-S, 853.2 μg/dL; reference range, 95.8–511.7). ACTH level was markedly elevated (963.7 pg/mL; reference range, 6.0–48.0), confirming ACTH-dependent CS. No dynamic hormonal testing was performed, considering the severe state of the patient. The hormonal findings are summarized in **Table 1B**.

**Table 1B:** Summary of initial hormonal assessment in presented patient.

Parameter	Value	Reference range
Morning serum cortisol (μg/dL)	78.2	3.7–19.4
Midnight serum cortisol (μg/dL)	69.1	< 5.4
mUFC (μg/24h)	11587.5	4.3–176.0
ACTH (pg/mL)	963.7	6.0–48.0
CgA (ng/mL)	13835.0	< 100.0
Urine metanephrines (μg/24h)	134.5	43.0–260.0
Urine normetanephrines (μg/24h)	189.3	128.0–484.0
Aldosterone (pg/mL)	43.2	25.2–392.0
DRC (µIU/mL)	5.2	4.4–46.1
DHEA-S (μg/dL)	853.2	95.8–511.7
Testosterone (ng/mL)	6.3	0.06–0.8
TSH (uIU/mL)	0.3	0.4–4.2
FT4 (pmol/L)	16.9	12.0–22.0
FT3 (pmol/L)	3.3	3.2–6.9

ACTH, adrenocorticotropic hormone; CgA, chromogranin A; DHEA-S, dehydroepiandrosterone sulfate; DRC, direct renin concentration; FT3, free triiodothyronine; FT4, free thyroxine; mUFC, mean urinary free cortisol; TSH, thyroid-stimulating hormone.

**Figure 1:**
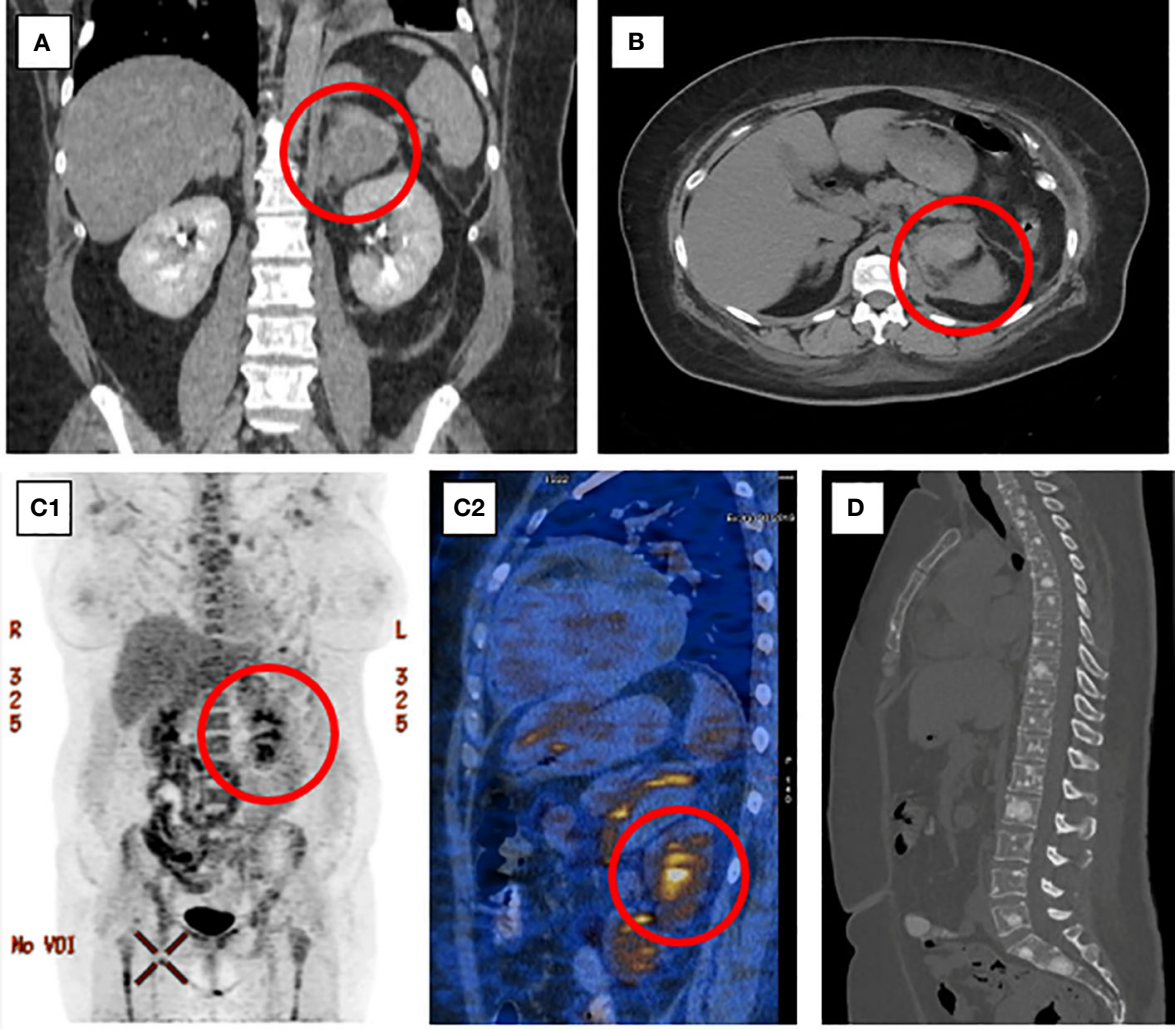
Frontal **(A)** and axial **(B)** CT scans of the abdomen presenting a large lesion of the left adrenal gland. 18F-FDG-PET-CT presenting high metabolic activity of the left adrenal gland lesion (C1, C2) and metastatic bone lesions in the spine and sternum **(D)**.

To control SH, continuous etomidate infusion was initiated with significant improvement in the patient’s general condition, edema reduction and normalization of blood pressure, glycemia, and potassium level with a decrease in the need for antihypertensive and insulin treatment, mineralocorticoid receptor blockade, and potassium supplementation. Pituitary magnetic resonance imaging (MRI) revealed no lesion. ACTH-dependent SH with negative pituitary imaging and short duration with rapid progression of symptoms were highly suggestive of EAS. Computed tomography (CT) of the chest, abdomen, and pelvis was performed and revealed a left, inhomogeneous, solid adrenal mass measuring 80 mm ×56 mm ×39 mm of 25 Hounsfield units adjacent to/infiltrating the left dome of the diaphragm, hyperplasia of the right adrenal gland, and numerous sclerotic bone lesions concerning for metastases (**Figure 1**).

Plasma and urine metanephrines, renin, and aldosterone levels were within the reference range (**Table 1B**). However, an excessively elevated concentration of chromogranin A (CgA) was observed (13,835.0 ng/mL; reference range, < 100), which firmly suggested the presence of a NET. The patient underwent whole-body SPECT-CT somatostatin receptor scintigraphy (SRS) with 99mTc-octreotate, which showed no evidence of somatostatin receptor overexpression. Subsequently, whole-body 18F-fluorodeoxyglucose (FDG)-positron emission tomography (PET)-CT was performed and revealed that the left adrenal gland lesion previously found on CT scan is partially metabolically active [maximum standardized uptake value (SUVmax), 3.2] and suspicious of malignancy; the right adrenal gland presented diffused 18F-FDG uptake (SUVmax, 2.6) likely resulting from excessive ACTH overstimulation. 18FDG-PET-CT also showed multifocal metabolically active sclerotic bone lesions in the spine, ribs, clavicles, scapules, sternum, pelvis, femurs, and humerus. A SUVmax 2.1 area (not correlated with CT imaging) was also found in the anterior mediastinum, which has been considered primarily as a residual thymus with physiological FDG uptake (**Figure 1**).

The clinical presentation was highly suggestive of generalized malignancy with ectopic ACTH secretion with the potential origin in the left adrenal gland. The patient was discussed at a multidisciplinary team meeting and was decided to undergo left-sided open adrenalectomy. After the surgery, the symptoms of hypercortisolemia resolved, the etomidate infusion could have been stopped, and the patient did not require further use of antihypertensive and insulin treatment, mineralocorticoid receptor blockade, and potassium supplementation. Furthermore, postoperatively, a significant decline in cortisol levels was noted (4.08 μg/dL), and the patient was transitioned to oral hydrocortisone. ACTH level also dropped (312.0 pg/mL 2 h after morning dose of oral hydrocortisone); however, it still remained significantly elevated. While waiting for the histopathological result, the patient underwent additional colonoscopy, gastroscopy, and bronchoscopy, but no other potential cancer origin was found. We consulted with the oncologist, and active surveillance was recommended until the histopathological examination results were obtained. The patient was discharged after 5 weeks of hospitalization, awaiting the result of the histopathological examination, in good general condition, requiring only hydrocortisone substitution.

The histopathological examination revealed an adrenal adenoma with the domination of adrenal infarction and necrosis. Immunohistochemistry (IHC) showed the following: CgA (+), EMA (−), synaptophysin (−), S100 (−), CKAE1/AE3 (+), RCC (−), melan-A (−), Ki-67 positive in single adrenal cells. Although its diagnosis was unlikely, according to the histopathology and IHC, it was not possible to clearly exclude the adrenocortical cancer (ACC). However, given ACTH-dependent hypercortisolemia and significantly elevated CgA concentration, an undetected neuroendocrine tumor was considered first.

**Figure 2:**
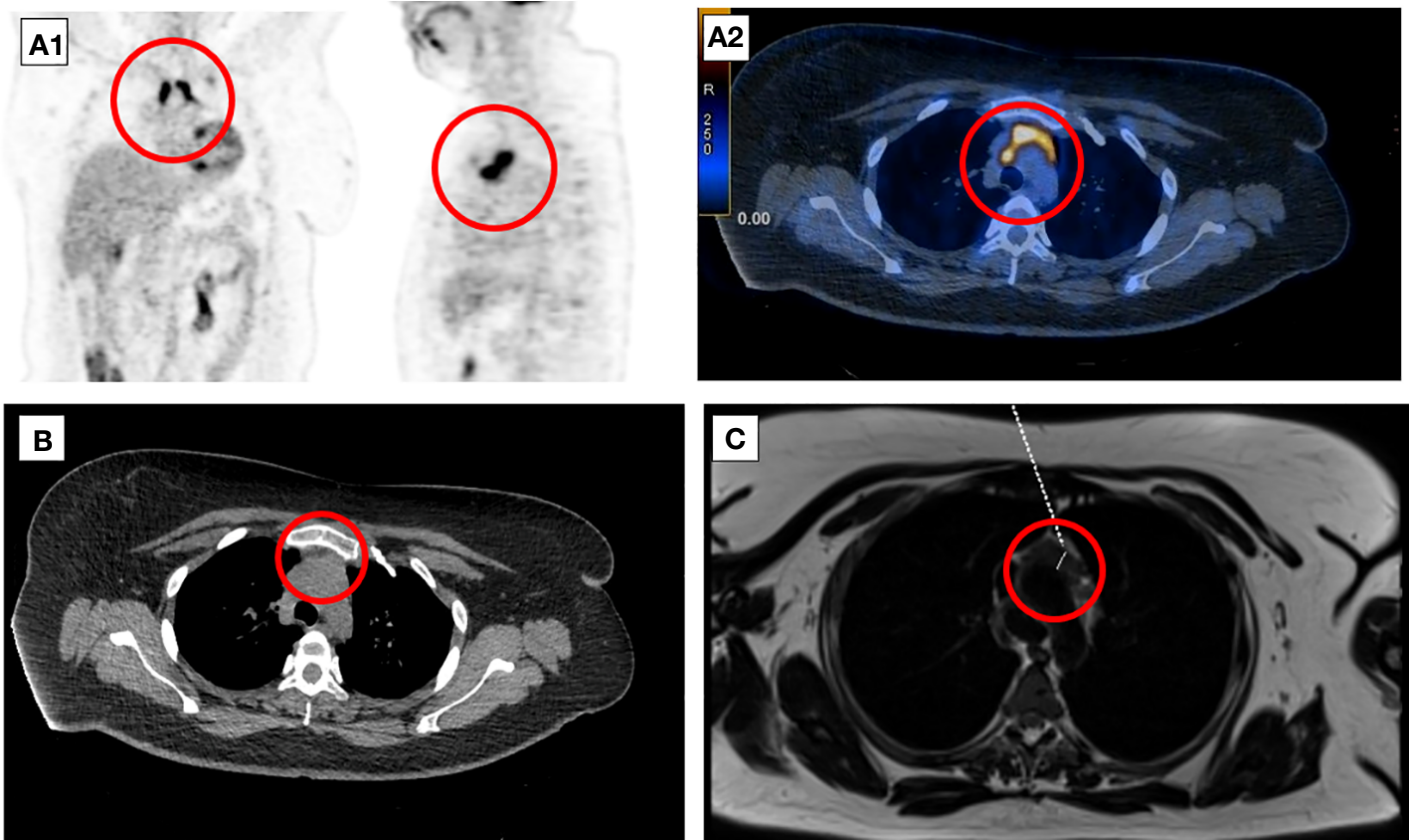
Eight-week follow-up 18F-FDG-PET-CT presenting metabolically active lesion in the superior anterior mediastinum in the thymus location (A1, A2). Axial CT **(B)** and MRI **(C)** scans of the chest presenting mediastinal mass corresponding to the invasive thymic malignancy.

A follow-up 18F-FDG-PET-CT performed after 8 weeks revealed a metabolically active mass (SUVmax, 9.3) in the superior anterior mediastinum in the thymus location (**Figure 2A**). CT (**Figure 2B**) and the subsequently performed MRI (**Figure 2C**) of the chest confirmed mediastinal mass measuring 42 mm × 33 mm, adjacent to the trachea and superior vena cava, encircling the ascending aorta and aortic arch, most likely corresponding to the invasive thymic malignancy.

**Figure 3:**
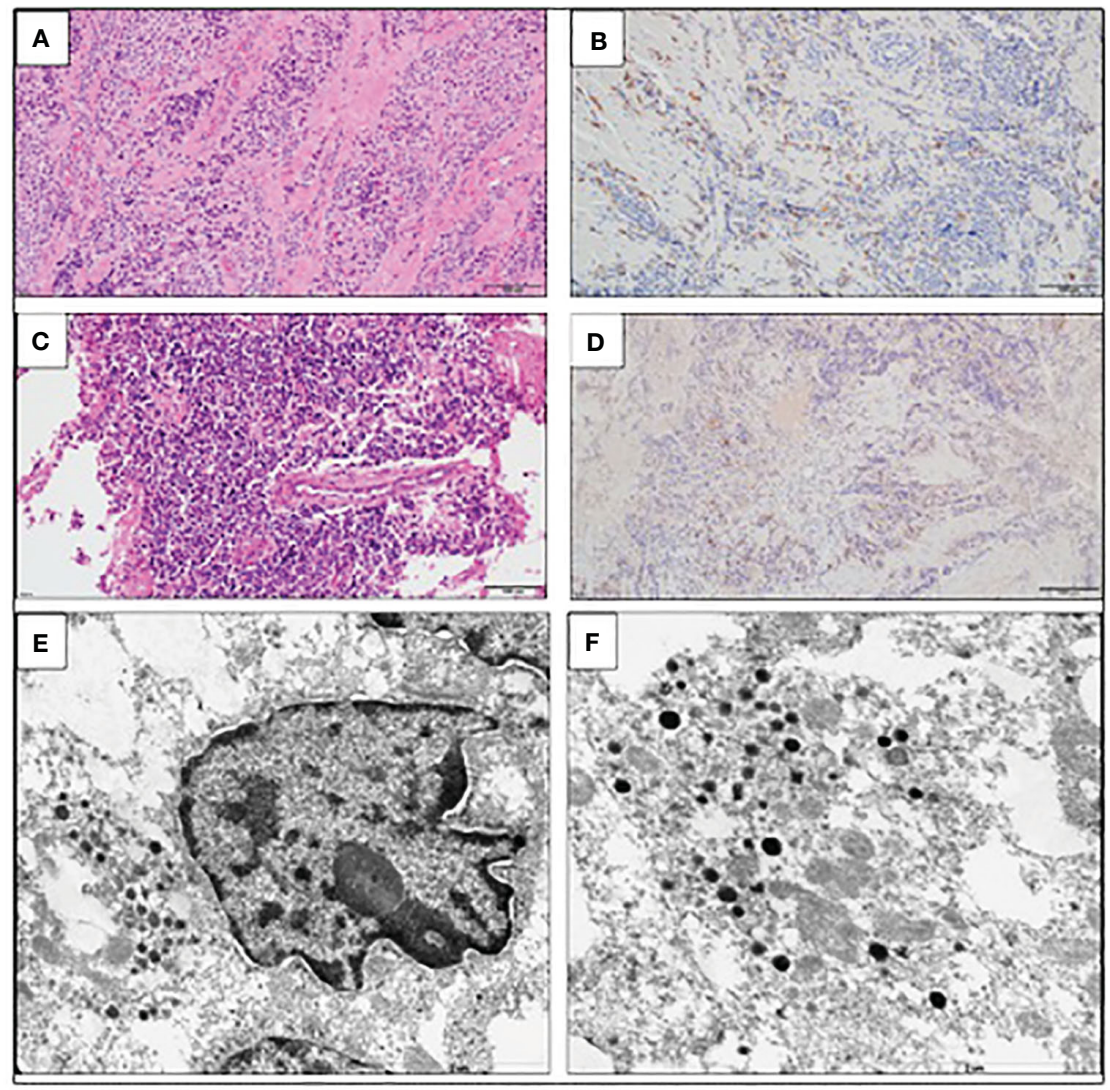
Large-cell neuroendocrine carcinoma of the thymus with atypical carcinoid morphology, pathological diagnosis. **(A)** Microscopic image of the primary tumor, HE staining. **(B)** Weak positive IHC staining for ACTH. **(C)** Microscopic image of the brain metastasis, HE staining. **(D)** Weak positive IHC staining for ACTH of the brain metastasis. **(E)** Electron microscopic image of cancer cells; the material was taken from a paraffin block, which resulted in a poorly preserved ultrastructure. Visible neuroendocrine granules concentrated around the cell nucleus (×17,500). **(F)** Ultrastructural image of the neuroendocrine granules with a diameter of (×24,500).

The patient was qualified for a thoracic surgery; however, due to the local invasion, it was only possible to perform a partial thymectomy (August 2019). After the procedure, the ACTH concentration dropped but not significantly (537.3 pg/mL before and 446.0 pg/mL after the surgery). A histopathology report revealed thymic LCNEC with AC component extensively infiltrating the surgical margins. On IHC, the tumor stained positive for CgA, synaptophysin, and CD56, and weakly for ACTH; the Ki-67 index was 40%, p53 expression was 70% (**Figure 3**). The final diagnosis was TNM stage IVB (pT2NxM1b), Masaoka–Koga stage III ACTH-secreting combined thymic LCNC and AC.

As part of the cancer multidisciplinary team meeting, the patient was qualified for postoperative radiotherapy (RTH) for the thymus bed and residual mass of the mediastinal tumor. Between October and December 2019, the patient received 64 Gy/t in 2 Gy/t fraction doses. Unfortunately, the ACTH concentration increased during the RTH to the maximum observed value of 1,021.2 pg/mL, which suggested further progression of the disease. Follow-up 18F-FDG-PET-CT (January 2020) showed a thymic mass measuring 31 mm × 20 mm, less metabolically active (SUVmax, 4.3) than initially. It also revealed a new 18F-FDG-avid lesion in the pancreas tail in the left temporal lobe (SUVmax, 12.7). The brain MRI confirmed the presence of metastasis measuring 30 × 24 × 18 mm. The patient was qualified for postoperative chemotherapy (CTH) according to the ADOC regimen (cisplatin, doxorubicin, vincristine, and cyclophosphamide) for aggressive thymic tumors (January 2020–May 2020). CTH resulted in disease partial response, and ACTH concentration dropped to 192.0 pg/mL. In June 2020, the patient underwent a craniotomy with left non-radical temporal tumor resection and received additional RTH. The disease was stable for almost 10 months; however, the follow-up 18F-FDG-PET-CT on May 2021 showed new active lesions in the right lung, pancreas, left iliopsoas muscle, and left breast. ACTH level at that time increased to 655.5 pg/mL. The patient was introduced to the PE regimen CTH (cisplatin and etoposide, June 2021–September 2021) with a short-term partial response. Because of the further disease progression, the patient was started on palliative CTH. More than 3.5 years after the first hospitalization, in November 2022, the patient passed away.

## 3 Discussion

In this paper, we present a unique case of a patient with ACTH-secreting combined thymic LCNEC with AC component, primary manifested as severe CS. NETTs constitute approximately 2%–5% of thymic tumors (7–9), representing approximately 2% of all mediastinal tumors (8). NETTS are typically diagnosed with a mean age of 55, with a clear male predominance (male-to-female ratio, 3:1) (7). EAS with ACTH secretion occurs in up to 50% of hormonally active NETTS (8). NETTs associated with EAS appear in younger populations below age of 40 compared to overall NETTs and are also more prevalent in male individuals (albeit in a lower proportion when compared to non-EAS NETTs) (9). EAS-related NETTs have a worse outcome than biochemically inactive thymic tumors, since they usually have an aggressive course, with early regional invasion, distant metastasis, and high mortality (8–11). Patients with EAS typically present with rapid-onset, severe CS, including resistant hypertension, hyperglycemia, profound and refractory hypokalemia with metabolic alkalosis, generalized edema, and proximal muscle weakness (4–6). SH, which occurs in approximately 60% of patients with hormonally active NETTs secreting ACTH (and approximately 80% in the case of ACTH-secreting thymic carcinomas), significantly worsens the initial poor prognosis (9). In the systematic review by Guerrero Pérez et al., mortality in patients with advanced disease was approximately 55%, and the median time between diagnosis and death was 38 months (9). NETTs are typically large tumors that could manifest with neoplastic mass effect (11, 12); however, only up to 10% of patients with ACTH-secreting NETTs present with local compressive symptoms (9).

In EAS, the progression of hypercortisolemia is typically accelerated, and patients with very rapid SH onset may not present with typical cushingoid features. During the initial assessment at the Emergency Department, the patient was overlooked—the physician’s attention was captured by diabetes mellitus, but the clinical features have not been linked to CS. It highlights the need for a high CS clinical suspicion in case of SH. The presence of profound hypokalemia in combination with hyperglycemia and resistant hypertension with edema is a clue that should prompt diagnosis (4, 5).

In the Endocrinology Department, the patient was quickly suspected of aggressive CS. Serum cortisol, UFC, and ACTH were dramatically increased. The patient was started on etomidate infusion to control hypercortisolemia. Etomidate is considered the most potent and effective agent for rapidly inhibiting cortisol overproduction (13, 14). Indeed, the patient’s clinical condition notably improved after only a few days of etomidate therapy with edema reduction and normalization of blood pressure, glycemia, and kalemia.

In the presented patient, the severity of hypercortisolemia with negative pituitary MRI image and positive whole-body CT imaging were compatible with EAS. Whole-body CT and functional imaging highly suggested a generalized malignancy with a potential origin in the left adrenal gland. The possibility of metastatic pheochromocytoma was considered—the concentration of CgA was significantly elevated, but urine and plasma metanephrines were negative. The ACC was also taken into consideration. Nevertheless, only one case of ACC potentially related to EAS was reported (15).

However, it is puzzling how only one of the adrenal glands was ACTH overstimulated, and the function of the second one seemed to be inhibited. A significant decline in cortisol concentration after exclusive unilateral adrenalectomy indicated that the left adrenal gland tumor could indeed have been the primary origin of malignancy and CS itself. On the other hand, ACTH level remained significantly elevated, although it dropped more than twofold compared to baseline. It was considered that the persistently elevated (but markedly lower) ACTH concentration was associated with the presence of metastases or could (albeit partially) result from the pituitary response to a significant decrease in cortisol concentration. Of course, metastatic ectopic ACTH-secreting tumor of unknown origin was also considered at that time.

Surprisingly, the histopathology examination revealed an adrenal adenoma with the domination of necrosis due to the adrenal infarction. It cannot be ruled out that the patient had a previously undiagnosed adrenal adenoma, and even short-term but dramatic ACTH hyperstimulation led it to its significant growth and provoked an adrenal infarction, imitating a malignancy in the imagery evaluation. Differentiating benign and malignant adrenal lesions based on 18F-FDG-PET-CT has a high diagnostic accuracy (16–18); however, metabolically active adenomas may present with increased FDG uptake and mimic malignancy (19). In addition, adrenal hemorrhage and necrosis can present with increased activity on 18F-FDG-PET-CT (20). Thus, it seems that the increased 18F-FDG avidity of the left adrenal lesion with foci of intratumoral necrosis was directly related to dramatically elevated ACTH concentrations and adrenal overstimulation.

CT scan located the EAS-related NETTs in 97.8% of cases in the aforementioned systematic review by Guerrero Pérez et al. (9). In the presented patient, imagery diagnostic initially failed to visualize the thymic tumor. There are only few reports on non-diagnostic chest CT or MRI evaluation in patients with EAS NETTs (21–23); however, in the presented cases, NETTs were found on SRS, which is contrary to our report. The first 18F-FDG-PET-CT localized an area of 2.1 SUVmax in the anterior mediastinum, initially considered a residual thymus because of the physiological FDG uptake. It is also unique how the PET-CT scan, chest CT, and MRI revealed a highly 18F-FDG-avid, large, invasive mediastinal mass just a few weeks after the baseline assessment. Imagery and functional studies were additionally retrospectively assessed by independent radiologists and nuclear medics to exclude a possible oversight during the initial analysis. However, it was maintained that there was no clear evidence of a thymic neoplasm at baseline.

Thymic carcinomas present with high FDG uptake, typically with SUVmax > 7 (24), SUV max values <4 as being most consistent with benign thymic processes (25). On the other hand, there is a marked overlap in FDG uptake between physiological thymic FDG uptake and thymic neoplasia in the literature, indicating that 18F-FDG-PET-CT has a limited ability to assess the thymus and an equivocal role in the differentiation of a normal thymus from thymic neoplasia (26). Among all described cases of EAS-related NETTs in the literature in which 18F-FDG-PET-CT was indicated as one of the diagnostic step, the primary tumor was visualized in all of them (27–38). The primary NETT SUVmax was reported only in five of them ranging from 2.48 to 12.0 (27–31); in the remaining ones, 18F-FDG avidity was reported from mild to high. However, in all mentioned cases (besides one (31), where no information about radiological chest imaging was reported), the NETT was previously visualized on chest CT, and 18F-FDG-PET-CT was performed to assess the disease staging rather than to find EAS origin.

On IHC, the thymic tumor stained weekly positive for ACTH in contrast to dramatically elevated plasma ACTH concentration. There seems to be a negative correlation between ACTH immunoreactivity and the neuroendocrine tumor malignancy potential. Moreover, the diagnosis of EAS is not ruled out in the case of primary tumor negative ACTH IHC staining. Less differentiated neuroendocrine tumors are believed to secrete ACTH rapidly and might also lose the ability to store ACTH in the secretory granules, thus leaving for typical techniques insufficient ACTH amounts stored to be stained (39, 40). In addition, the tumor might secrete various biologically active ACTH precursors that are negative on IHC.

Complete NETT resection is the only curative option and the strongest factor for overall survival (7, 41, 42). In the case of a subtotal resected tumor, RTH and CTH are considered (42–44); however, there is no consensus and guidelines for the optimal postoperative strategy, mainly due to the rarity of the disease. Systematic therapies are also used as palliative treatment in case of unresectable, metastatic, and recurrent NETTs (42, 44). Besides the primary metastatic disease, the presented patient underwent a partial resection with macroscopic residual tumor (R2) and was then qualified for postoperative RTH and CTH. After the R2 resection, postoperative RTH may be combined sequentially or concurrently with CTH (45). RTH was not clearly effective, as the follow-up 18F-FDG-PET-CT showed new metastases, including an extensive metastasis to the left temporal lobe. Several CTH regimens have been used in patients with NETTs (42, 44, 45). CTH response rates in metastatic poorly differentiated NETTs are 30%–50%, with progression-free survival rates of 6–9 months (45). The presented patient was introduced to an ADOC regimen, which resulted in a partial response. However, the patient started second-line and later palliative CTH because of the further disease progression.

## 4 Conclusions

We present a unique and challenging case of malignant, primary metastatic NETT initially manifesting with severe EAS and not visible in initial imaging studies in a patient with coexisting adrenal tumor suspected of malignancy and primary disease origin. The presented case highlights that the diagnosis and management of EAS remain challenging; it requires a high clinical suspicion, rapid hypercortisolemia control with symptomatic treatment of cortisol-induced comorbidities, and simultaneously complex imaging studies to determine the primary source of the ACTH hypersecretion. The treatment of choice is resection of ACTH-secreting NET; however, it may not be possible in patients with initially occult or metastatic disease. Malignant NETTs with ectopic CS are extremely rare, and their management has to be individualized in every case, requiring a multidisciplinary approach. Regardless, the prognosis remains poor due to the aggressiveness of the disease.

## Data availability statement

The original contributions presented in the study are included in the article/supplementary material. Further inquiries can be directed to the corresponding author.

## Ethics statement

The studies were conducted in accordance with the local legislation and institutional requirements. The patient gave an oral consent for publication while alive. Written informed consent for publication was obtained from the patient’s parents.

## Author contributions

LD: Resources, Writing – review & editing, Writing – original draft, Methodology, Investigation, Formal analysis, Data curation, Conceptualization. AW-L: Writing – review & editing, Supervision, Resources, Methodology, Data curation, Conceptualization. MM: Writing – review & editing, Resources, Data curation, Conceptualization. PW: Writing – review & editing, Supervision, Resources, Methodology, Data curation.
